# Analysis of the Influence of Thyroid Nodule Characteristics on the Results of Shear Wave Elastography

**DOI:** 10.3389/fendo.2022.858565

**Published:** 2022-06-10

**Authors:** Ji-ping Xue, Xiao-yan Kang, Jun-wang Miao, Yan-xia Zhang, Hui-zhan Li, Fu-cheng Yao, Chun-song Kang

**Affiliations:** Department of Ultrasonography, Shanxi Bethune Hospital, Shanxi Academy of Medical Sciences, Tongji Shanxi Hospital, The Third Hospital of Shanxi Medical University, Taiyuan, China

**Keywords:** thyroid, nodule characteristics, shear wave elastography, influence, ultrasonography

## Abstract

**Objective:**

To analyze the ultrasonic characteristics of false-negative and false-positive results of shear wave elastography (SWE) in the diagnosis of thyroid nodules to clarify the influence of nodular characteristics on SWE and to guide the clinical application of SWE.

**Methods:**

A total of 435 thyroid nodules from 343 patients with the diagnosis confirmed by surgical pathology were analyzed. Preoperative ultrasonography and SWE were conducted. The conventional ultrasound characteristics of thyroid nodules and the maximum Young’s modulus were recorded. The false negativity and false positivity of SWE for the diagnosis of thyroid nodules were calculated. The ultrasonic characteristics of thyroid nodules with SWE false results were analyzed, and logistic regression analysis was adopted to determine the ultrasonic characteristics associated with SWE false results of thyroid nodules.

**Results:**

Among 323 malignant nodules, the SWE false negativity was 27.2% (88/323). The false positivity of SWE in 112 benign nodules was 19.6% (22/112). Regression analysis showed that an increase in the nodule volume increased the risk of SWE false-positive results (odds ratio [OR] 3.286; 95% confidence interval [CI]: 1.572–6.871; P = 0.002) and decreased the risk of false-negative results (OR 0.238; 95% CI: 0.115–0.493; P < 0.001). Nodules with coarse calcification had an increased risk of SWE false-positive results compared with those without calcification (OR 5.303; 95% CI: 1.098–25.619; P = 0.038). However, nodules with scattered hyperechoic foci had a reduced risk of SWE false-negative results (OR 0.515; 95% CI: 0.280–0.951; P = 0.034).

**Conclusion:**

Nodular size and calcification were correlated with SWE false results, and the clinical application of SWE should be combined with conventional ultrasound features. Fine needle aspiration or a puncture biopsy should be conducted if necessary.

## Introduction

Thyroid nodules are a common clinical problem that 19%–68% of the population suffer from, and thyroid cancers account for 7%–15% among these (1). Ultrasound is the first choice to determine the nature of thyroid nodules. Morphological characteristics are an important basis for differentiating between benign and malignant thyroid nodules. In addition, changes in tissue texture are closely correlated with the pathological development of thyroid nodules (2).

Shear wave elastography (SWE) is a new method to qualitatively and quantitatively evaluate tissue hardness (3) and is suggested to be used as a supplement to conventional ultrasound to determine the nature of thyroid nodules, rather than replacing conventional ultrasound as a separate diagnostic tool. Accumulating studies have shown that SWE has a certain value in distinguishing benign and malignant thyroid nodules, but no widely applicable diagnostic criteria have been established, which is mainly due to the fact that the results of SWE will be affected by many factors (4, 5).

The histological characteristics of nodules may affect the results of SWE (6), and the sonographic features can, to some extent, reflect the characteristics of nodules. A comparative analysis of the nodular features of misdiagnosed and correctly diagnosed nodules by SWE and the identification of which features are likely to produce incorrect results will help to determine the accuracy of SWE results. Therefore, in order to guide the clinical application of SWE, this study investigated the impact of nodular features on SWE through the analysis of SWE in the diagnosis of thyroid nodules in the false negative and false positive results of the relevant sonographic features.

## Materials and Methods

### General Data

From June 2016 to July 2018, 343 patients with 435 thyroid nodules were enrolled in the study. All the patients underwent thyroid surgery and had pathological results. There were 78 males and 265 females aged from 24 to 77 years, with an average age of 47.9 ± 10.5 years. The maximum diameter of the nodules ranged from 3.0 mm to 57.0 mm, with an average of 11.0 ± 7.7 mm.

The inclusion criteria were as follows: (1) patients aged >18 years old without treatment or puncture before the ultrasound examination; (2) patients who underwent two-dimensional ultrasound and SWE ultrasound examination before surgery.

The exclusion criteria were as follows: (1) patients with cystic changes in the nodules >25%; (2) patients with loss of elastic image information because of coarse calcification in or around the nodules; (3) patients with nodules in the isthmus or adjacent to the trachea cartilage or the *arteria carotis communis*, since the lateral displacement of the pulsing may cause artifacts in the hardness of the nodules; (4) patients with benign and malignant nodules in the same thyroid lobe.

### Research Method

A SuperSonic Imagine Aixplorer^®^ diasonograph made in France was used for thyroid conventional ultrasound and SWE with the linear probe set at a frequency of 4–15 MHz or 2–10 MHz. All images were collected by the same sonographer, who had more than 10 years of experience.

Conventional ultrasound examination: The patients were maintained in a supine position with full exposure of the neck. The probe was placed lightly on the neck without pressure. The patients could not move or swallow during the examination. The nodule features observed were (1) size; (2) composition: mixture, solid; (3) echo: hyperechoic or isoechoic, hypoechoic, extremely hypoechoic; (4) shape: an anteroposterior/transverse diameter (AP/T) ratio >1, AP/T ratio <1; (5) edge: clear, lobed, or irregular extraglandular invasion; (6) calcification: microcalcification, extensive calcification, no calcification. All nodules were scored according to the American College of Radiology Thyroid Imaging Reporting and Data System (ACR–TIRADS) (7).

Shear wave elastography: After a conventional ultrasound examination, the target nodule was identified on the longitudinal section of the thyroid gland, and the instrument was switched to the SWE mode (with a scale plate of 0–100 kPa). A sampling frame with the inclusion of the entire lesion and the surrounding normal thyroid tissue was placed on the nodule, and the patient was asked to hold their breath and stand for 3–5 s. Once stabilized, the image was frozen and an elastic image was obtained. On the image, blue represented the relatively soft tissue and red represented the relatively hard tissue. The circular region of interest (Q–Box™) was placed at the hardest part of the solid nodules with a diameter of 2–4 mm to avoid visible calcification. The maximum elastic value (Emax), minimum elastic value (Emin), and the average elastic value (Emean) of each nodule were automatically generated. Each nodule was measured three times, and the average value was calculated.

### Analysis Indicators

The characteristics of SWE in the diagnosis of thyroid nodules were compared between the group with false positivity and the group with false negativity, and the related factors leading to false-negative and false-positive results were analyzed.

### Statistical Analysis

The SPSS^®^ Statistics 22.0 statistical software was used for statistical analysis, and the pathological results were taken as a reference standard to construct the receiver operating characteristic (ROC) curves of Emax, Emean, and Emin. The area under the curve (AUC) of each parameter was calculated. The parameter with the highest AUC value was taken as the SWE diagnostic parameter, and the optimal cut-off point was taken as the diagnostic criterion. In pathologically malignant nodules, those with an SWE diagnostic parameter greater than the diagnostic criterion were classified as the true-positive group, and those with an SWE diagnostic parameter less than the diagnostic criterion were classified as the false-negative group. In pathologically benign nodules, those with an SWE diagnostic parameter less than the diagnostic criterion were classified as the true-negative group, and those with an SWE diagnostic parameter greater than the diagnostic criterion were classified as the false-positive group.

The measurement data that satisfied the normal distribution were expressed as means ± standard deviations, and the differences between groups were compared by the independent sample t-test. The measurement data that did not satisfy the normal distribution were represented by medians and inter-quartile ranges and analyzed by the nonparametric rank-sum test. The enumeration data were analyzed by the chi-squared test or Fisher’s exact test. Multivariate analysis of the SWE false results was conducted by logistic regression analysis; P < 0.05 was considered statistically significant.

## Results

### Pathologic Results

A total of 343 patients had 435 nodules, including 112 (25.7%) benign nodules. There were 103 cases with nodular goiter, 6 cases with adenoma, 1 case with subacute thyroiditis, and 2 cases with Hashimoto’s thyroiditis. There were 323 (74.3%) malignant nodules, including 318 papillary carcinomas, 3 follicular adenocarcinomas, and 2 medullary carcinomas.

### Diagnostic Performance of the SWE Parameters

The SWE parameters Emax, Emean, and Emin of the malignant nodules were significantly higher than those of the benign ones (P < 0.001 in all) (**Table 1**). Among the quantitative parameters of SWE, when Emax was at the optimal cut-off point of 36.15 kPa, the AUC reached 0.818 (**Table 2**), with a sensitivity of 72.8%, a specificity of 80.4%, a positive predictive value of 91.4%, a negative predictive value of 50.6%, and 74.7% accuracy.

**Table 1:** Comparison of the SWE quantitative parameters between the benign and malignant thyroid nodules.

Parameter	Malignant nodules	Benign nodules	Z	*P*
Emax(kpa)	24.6 (18.3, 34.3)	49.4 (34.4, 69.6)	-10.043	<0.001
Emean(kpa)	18.6 (13.8, 25.2)	35.4 (24.8, 51.1)	-9.910	<0.001
Emin(kpa)	13.0 (9.0, 17.8)	20.7 (13.3, 31.3)	-6.788	<0.001

Note: the data was represented as the median and inter-quartile range.

**Table 2:** Diagnostic efficiency of the SWE parameters.

Parameters	Cut-off	Sensitivity	Specificity	Accuracy	Negative predictive value	Positive predictive value	Area under the curve
Emax	>36.15Kpa	72.80%	80.40%	74.70%	50.60%	91.40%	0.818 (0.774-0.863)
Emean	>29.85Kpa	63.80%	88.40%	70.10%	45.80%	94.10%	0.814 (0.771-0.858)
Emin	>18.05Kpa	60.70%	75.00%	64.40%	39.80%	87.50%	0.715 (0.666-0.765)

### Univariate Analysis of the Factors Influencing the Diagnostic Results of SWE

**Figure 1:**
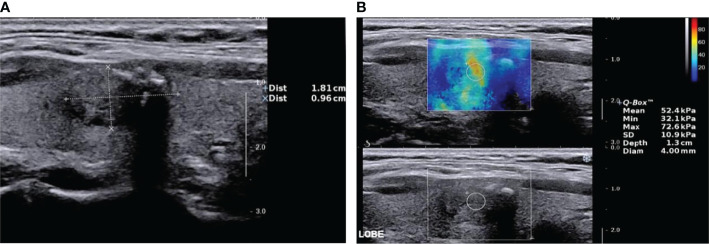
Nodular goiter (shear wave elastography false-positive nodule) by two-dimensional ultrasound and elastic ultrasound. **(A)** A solid isoechoic nodule in the middle of the right lobe of the thyroid, 18.1 × 9.6 mm, with an ill-defined margin and coarse calcification. The ACR–TIRADS score was 4 and ACR–TIRADS category was 4. **(B)** The Emax value of the nodule on SWE imaging was 72.6 kPa.

When “Emax > 36.15 kPa” was used as the standard for a positive result for SWE, 19.6% (22/112) of the benign nodules had an Emax greater than 36.15 kPa and were classified in the false-positive group (**Figure 1**), while 80.4% (90/112) of the benign nodules had an Emax less than 36.15 kPa and were classified in the true-negative group (**Table 3**). The sonographic findings of the nodule size, edge, and calcification were correlated with the SWE false-positive results (P < 0.05). In the ACR–TIRADS classification, the proportion of nodules with an ACR–TIRADS grade of 1–3 in the benign nodule false-positive group (22.7%, 5/22) was significantly lower than that in the true-negative group (60.0%, 54/90) (P = 0.021).

**Table 3:** Comparison of the related characteristics of thyroid nodule for diagnosis by SWE.

		Malignant nodules n(%)				Benign nodules n(%)			
		False negative (n=88)Emax ≤ 36.15Kpa	True positive(n=235)	Statistics	*P*	False positive(n=22)	True negative(n=90)	Statistics	*p*
			Emax>36.15 Kpa			Emax>36.15 Kpa	Emax ≤ 36.15Kpa		
Gender	Female	71(80.7)	184(78.3)	χ^2^ = 0.219	0.64	17(77.3)	71 (78.9)	χ^2^ = 3.696	0.055
Age	(year)	46.8 ± 8.2	45.7 ± 10.5	*t*=-0.894	0.372	53.3 ± 10.4	53.4 ± 10.5	*t*=0.019	0.985
The largest diameter	(cm)	0.60 (0.40, 0.80)	0.90(0.70, 1.50)	Z=-6.989	<0.001	1.55 (0.98, 2.15)	0.80 (0.60, 1.33)	Z=-3.029	0.002
Nodule size	<1cm	78 (88.6)	132(56.2)	χ^2^ = 31.072	<0.001	6 (27.3)	52 (57.8)	χ^2^ = 9.599	0.007
	1cm-2cm	10 (11.4)	76 (32.3)			8 (36.4)	28 (31.1)		
	>2cm	0 (0)	27 (11.5)			8 (36.4)	10 (11.1)		
Structure	Spongy	0 (0)	0 (0)	χ^2^ = 0.006	0.936	0 (0)	5 (5.6)	χ^2^ = 2.480	0.249
	Solid-cystic	2 (2.3)	5(2.1)			2 (9.1)	19 (21.1)		
	Solid	86 (97.7)	230 (97.9)			20 (90.9)	66 (73.3)		
Echo	Echo or hyperechoic	3 (3.4)	14 (6.0)	χ^2^ = 1.263	0.545	10 (45.5)	54 (60.0)	χ^2^ = 1.527	0.217
	Hypoechoic	81 (92.0)	214 (91.0)			12 (54.5)	36 (40.0)		
	Extremely hypoechoic	4(4.6)	7 (3.0			0 (0)	0 (0)		
Edge	Smooth	21 (23.9)	29 (12.3)	χ^2^ = 15.603	<0.001	15 (68.2)	80 (88.9)	χ^2^ = 4.839	0.036
	Irregular	67 (76.1)	179 (76.2)			7 (31.8)	10 (11.1)		
	Extrathyroidal invasion	0 (0)	27 (11.5)			0 (0)	0 (0)		
Anteroposterior/transverse diameter ratio	<1	28 (31.8)	105 (44.7)	χ^2^ = 4.373	0.037	21 (95.5)	81 (90.0)	χ^2^ = 0.150	0.699
	>1	60(68.2)	130 (55.3)			1 (4.5)	9 (10.0)		
Calcification	Without calcification	57 (64.8)	88 (37.4)	χ^2^ = 20.634	<0.001	12 (54.5)	78 (86.7)	χ^2^ = 10.671	0.004
	Coarse calcification	9 (10.2)	27 (11.5)			4 (18.2)	5 (5.5)		
	scattered hyperechoic foci	22 (25.0)	120 (51.1)			6 (27.3)	7 (7.8)		
TI-RADS classification	Class 1	0 (0)	0 (0)	χ^2^ = 6.904	0.053	0 (0)	5 (5.6)	χ^2^ = 10.526	0.021
	Class 2	1 (1.1)	1 (0.4)			1 (4.5)	12 (13.3)		
	Class 3	2 (2.3)	1 (0.4)			4 (18.2)	37 (41.1)		
	Class 4	17 (19.3)	27 (11.5)			10 (45.5)	27 (30.0)		
	Class 5	68 (77.3)	206 (87.7)			7 (31.8)	9 (10.0)		

**Figure 2:**
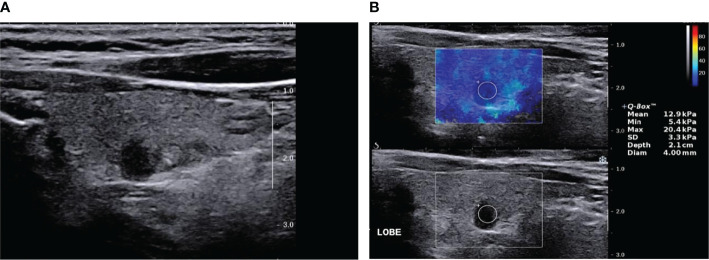
Papillary thyroid carcinoma (SWE false-negative nodule) by two-dimensional ultrasound and elastic ultrasound. **(A)** A solid, very hypoechoic nodule in the middle-left lobe of the thyroid, 6.0 × 3.0 mm, with an ill-defined margin and taller than wide in shape. The ACR-TIRADS score was 8 and ACR–TIRADS category was 5. **(B)** The Emax value of the nodule on SWE imaging was 20.4 kPa.

When “Emax > 36.15 kPa” was used as the standard for a positive result for SWE, 27.2% (88/323) of the malignant nodules had an Emax less than 36.15 kPa and were classified in the false-negative group (**Figure 2**), while 72.8% (235/323) of the malignant nodules had an Emax greater than 36.15 kPa and were classified in the true-positive group (**Table 3**). Nodule size, edge, AP/T ratio, and calcification were correlated with the SWE false-negative results (P < 0.05). In the ACR–TIRADS classification, there was no significant difference in the proportion of nodules with an ACR–TIRADS grade of 4–5 between the false-negative group (96.6%, 85/88) and the true-positive group (99.2%, 233/235) (P = 0.053).

### Multivariate Analysis of the Factors Influencing the Diagnostic Results of SWE

Multiple logistic regression analysis of the factors correlated with the false-positive results in SWE: Multiple logistic regression equations were established by incorporating the nodule size, edge, and calcification. The results suggested that the risk of SWE false-positive results increased with an increase in the nodule volume (OR 3.286; 95% CI: 1.572–6.871; P = 0.002). Nodules with coarse calcification had an increased risk of false-positive SWE results compared with those without nodular calcification (OR 5.303; 95% CI: 1.098–25.619; P = 0.038) (**Table 4**).

**Table 4:** Multiple logistic regression analysis for the SWE false positive results.

Factor	Group	B	SE	Wald chi-square value	*P*	*OR*	95% CI
Nodule size		1.190	0.376	9.995	0.002	3.286	1.572~6.871
Edge	Smooth*						
	Irregular	1.298	0.726	3.198	0.074	3.661	0.883~15.179
Calcification	Without calcification*						
	Coarse calcification	1.668	0.804	4.310	0.038	5.303	1.098~25.619
	Microcalcification	1.143	0.763	2.244	0.134	3.137	0.703~14.000

*The control group.

Multiple logistic regression analysis of the factors correlated with the false-negative results in SWE: Multiple logistic regression equations were established by incorporating the nodule size, edge, AP/T ratio, and calcification. The results suggested that the risk of SWE false-negative results decreased with an increase in the nodule volume (OR 0.238; 95% CI: 0.115–0.493; P < 0.001). Conversely, the smaller the nodule volume, the greater the risk of SWE false-negative results. Compared with the nodules without calcification, nodules with scattered hyperechoic foci had a reduced risk of SWE false-negative results (OR 0.51; 95% CI: 0.280–0.951; P = 0.034) (**Table 5**).

**Table 5:** Multivariate logistic regression analysis for the SWE false negative results.

Factor	Group	B	SE	Wald chi-square value	*P*	*OR*	95% CI
Nodule size		-1.436	0.372	14.925	<0.001	0.238	0.115~0.493
Edge	Smooth*						
	Irregular	-0.695	0.363	3.667	0.055	0.499	0.245~1.016
Anteroposterior/transverse diameter ratio		-0.024	0.108	.049	0.826	0.977	0.791~1.206
Calcification	Without calcification*						
	Coarse calcification	-0.569	0.456	1.553	0.213	0.566	0.232~1.385
	Micro calcification	-0.663	0.312	4.503	0.034	0.515	0.280~0.951

*The control group.

## Discussion

Many studies have shown that SWE is valuable in diagnosing the nature of thyroid nodules by evaluating the inherent characteristics of thyroid nodules. However, there are still some problems in the diagnosis of SWE, such as the large overlap of the elastic values of some benign and malignant nodules, and the wide range of cutoff values for the diagnosis of thyroid malignant nodules, ranging from 26 to 85kPa (8–10), which may be related to the great heterogeneity between different studies or between nodules. Swan et al. (6) reported that the diagnostic efficacy of SWE at the individual level is not satisfactory, so it seems difficult to establish a clear and widely applicable diagnostic criteria in the short term. However, the clinical application of SWE is for individual patients, so it is particularly important to interpret the reliability of SWE results. Therefore, by comparing the sonographic characteristics of false negative and false positive results of SWE in the diagnosis of thyroid nodules, and analyzing the influence of ultrasound characteristics of thyroid nodules on SWE, this study helps to judge whether the results of SWE are reliable. In this study, the ROC curve was used to determine the best diagnostic index Emax. When 36.15kPa, the cutoff value of Emax, was used as the diagnostic criteria, the false positive rate of benign nodules was 19.6% (22cm/112) and the false negative rate of malignant nodules was 27.2% (88/323).

Shang et al. (11) showed that the internal composition of nodules changes with the increase of nodular volume, and the hardness increases. The results of the present study show that the size of nodules impacted the SWE results. The greater the nodule volume, the higher the risk of SWE false-positive results, and the lower the risk of false-negative results. Therefore, too large or too small nodules will affect the diagnostic efficacy of SWE, which is consistent with the research results of Wang et al. (12) Elastography has a low sensitivity for nodules smaller than 10 mm and low specificity for nodules larger than 20 mm. The components of benign and malignant thyroid nodules change during their development. When malignant nodules are small, the fibrous components are relatively small with low hardness, but as they increase in volume, fiber composition increases and calcification may occur. As benign nodules increase in volume, extensive fibrosis and hyaline changes occur, both of which will cause an increase in the nodular hardness (13, 14).

In addition, calcification has been thought to be an important factor in increasing the hardness value. Among malignant nodules in the present study, the proportion of nodules with scattered hyperechoic foci in the true-positive group (51.1%, 120/235) was significantly higher than that in the false-negative group (25.0%, 22/88). Multivariate regression analysis showed that compared with the non-calcified nodules, nodules with scattered hyperechoic foci had a lower risk of SWE false-negative results. In the benign nodules, the percentages of coarse calcification (18.2%, 4/22) and scattered hyperechoic foci (27.3%, 7/22) in the false-positive group were significantly higher than those in the true-negative group (5.6%, 5/90; and 7.8%, 7/90, respectively). Multivariate regression analysis showed that coarse calcification was a risk factor for the SWE false-positive results. This is consistent with the results of Gregory et al. (15), which meant that cluster microcalcification and coarse calcification might lead to a significant increase in the hardness value. The scattered hyperechoic foci in the benign nodules was not a risk factor for the SWE false-positive results, possibly because part of the scattered hyperechoic foci in the benign nodules was not real calcification but concentrated colloid or fibrous tissue (16). The effect on the SWE value was relatively small; therefore, attention should be paid to identification in clinical application.

The results of multivariate analysis in the present study showed that the AP/T ratio and edge had no significant correlation with the SWE false results. The AP/T ratio is the ratio of the anterior and posterior diameter to the transverse diameter of nodules, reflecting the growth mode of nodules, while SWE mainly reflects the internal characteristics of nodules (13). An AP/T ratio >1 is considered an important sign of thyroid malignant nodules in conventional ultrasound, especially for nodules smaller than 10 mm, with a high sensitivity (81.4%) and specificity (96.8%) for the diagnosis of malignant nodules (17). Therefore, for nodules smaller than 10 mm, the combination of two-dimensional ultrasonic characteristics may be more helpful for judging the nature of nodules. Yoo et al. (18) found that the combination of the Emax value of nodules and the aspect ratio information of nodules is not only valuable in distinguishing benign from malignant nodules, but also helpful to predict the pathological types of thyroid nodules. However, two-dimensional ultrasound is of limited value when large, benign nodules with coarse calcification present a high risk of a false-positive result in SWE. In the present study, the proportion of nodules with an ACR–TIRADS grade of 4–5 in the benign nodules’ SWE false-positive group (77.3%, 17/22) was significantly higher than that in the true-negative group (40.0%, 36/90). Therefore, for nodules with an ACR–TIRADS score of Class 4 or above, fine needle aspiration or biopsy is necessary for further diagnosis.

### Limitations

This study only analyzed the correlation between the sonographic features of nodules and the results of SWE diagnosis, without considering other related factors, such as nodular location, depth and surrounding tissue. Moreover, this study is a retrospective study with a high proportion of malignant nodules and a small number of benign nodules, and there is a certain selection bias. Finally, the malignant nodules in this study are mainly papillary carcinoma, and other pathological properties such as follicular carcinoma and medullary carcinoma need to be further studied.

## Conclusion

when the volume of malignant nodules is less than 10mm, SWE is predisposed to produce false negative results, and combining two-dimensional ultrasound features is more helpful to improve the diagnostic accuracy. For nodules with clustered microcalcification, the risk of false negative is reduced. For benign nodules with large volume and coarse calcification, SWE has a higher risk of producing false positive results, and further diagnosis by FNA or fine needle biopsy is needed if necessary. Therefore, this study has a certain guiding significance in defining the scope and value of clinical application of SWE.

## Data Availability Statement

The original contributions presented in the study are included in the article/supplementary material. Further inquiries can be directed to the corresponding author.

## Ethics Statement

The studies involving human participants were reviewed and approved by The Third Hospital of Shanxi Medical University, Shanxi Bethune Hospital, Shanxi Academy of Medical Sciences. The patients/participants provided their written informed consent to participate in this study.

## Author Contributions

Conception and design of the research: C-sK, J-pX; Acquisition of data: J-wM and Y-xZ; Analysis and interpretation of the data: X-yK and H-zL; Statistical analysis: X-yK, J-wM, and F-cY; Obtaining financing: J-pX. Writing of the manuscript: J-pX. Critical revision of the manuscript for intellectual content: C-sK. All authors read and approved the final draft.

## Funding

Key research and Development Projects in Shanxi Province (grant number 201803D31143).

## Conflict of Interest

The authors declare that the research was conducted in the absence of any commercial or financial relationships that could be construed as a potential conflict of interest.

## Publisher’s Note

All claims expressed in this article are solely those of the authors and do not necessarily represent those of their affiliated organizations, or those of the publisher, the editors and the reviewers. Any product that may be evaluated in this article, or claim that may be made by its manufacturer, is not guaranteed or endorsed by the publisher.
